# Enhancing Occupant Comfort and Building Sustainability: Lessons from an Internet of Things-Based Study on Centrally Controlled Indoor Shared Spaces in Hot Climatic Conditions

**DOI:** 10.3390/s24051406

**Published:** 2024-02-22

**Authors:** Parag Kulkarni, Bivin Pradeep, Rahemeen Yusuf, Henry Alexander, Hesham ElSayed

**Affiliations:** 1 College of Information Technology (CIT), United Arab Emirates University, Al Ain P.O. Box 15551, United Arab Emirates; bivinz2013@uaeu.ac.ae (B.P.); 201990006@uaeu.ac.ae (H.A.); helsayed@uaeu.ac.ae (H.E.); 2 National Water and Energy Centre (NWEC), United Arab Emirates University, Al Ain P.O. Box 15551, United Arab Emirates; 3 Emirates Centre for Happiness Research, United Arab Emirates University, Al Ain P.O. Box 15551, United Arab Emirates; r.yusuf@uaeu.ac.ae

**Keywords:** Internet of Things (IoT), occupant comfort, building sustainability, energy efficiency

## Abstract

It is well known that buildings have a sizeable energy and environmental footprint. In particular, in environments like university campuses, the occupants as well as occupancy in shared spaces varies over time. Systems for cooling in such environments that are centrally controlled are typically threshold driven and do not account for occupant feedback and thus are often relying on a reactive approach (fix after identifying problems). Therefore, having a fixed thermal operating set point may not be optimal in such cases—both from an occupant comfort and well-being as well as an energy efficiency perspective. To address this issue, a study was conducted which involved development and deployment of an experimental Internet of Things (IoT) prototype system and an Android application that facilitated people engagement on a university campus located in the UAE which typically exhibits hot climatic conditions. This paper showcases data driven insights obtained from this study, and in particular, how to achieve a balance between the conflicting goals of improving occupant comfort and energy efficiency. Findings from this study underscore the need for regular reassessments and adaptation. The proposed solution is low cost and easy to deploy and has the potential to reap significant savings through a reduction in energy consumption with estimates indicating around 50–100 kWh/day of savings per building and the resulting environmental impact. These findings would appeal to stakeholders who are keen to improve energy efficiency and reduce their operating expenses and environmental footprint in such climatic conditions. Furthermore, collective action from a large number of entities could result in significant impact through this cumulative effect.

## 1. Introduction

Dwindling hardware costs, small form factor devices, widespread connectivity, and advances in data management and analytics have created a potent combination which is being leveraged to solve real world problems. One of the pressing issues facing the world at large is reducing impacts on the environment. This has prompted nations to commit to net zero carbon emission pledges. Achieving net zero broadly involves reaching an equilibrium between the energy consumed and an equal amount of it being generated through sustainable alternatives to offset consumption. Whilst there are many different approaches to achieving this both on the demand and supply side, the focus in this paper is on one potential pathway that involves identifying ways in which energy efficiency can be improved through IoT-driven interventions. In the context of this work, the focus is on a university campus environment.

University campuses comprise large numbers of buildings with a sizeable energy footprint. One of the primary contributors to the energy footprint of a building is the Heating Ventilation Air Conditioning (HVAC) system which is used to heat/cool the building. Therefore, opportunities to optimise its operation have the potential for significant benefit. Whilst this is important from an operational perspective, it is also equally important to ensure that one of the primary stakeholders—the people in the buildings—are comfortable and happy. Therefore, understanding people’s experiences of their comfort level is crucial. Often, building managers set a target operating point which is generally considered as ‘acceptable’ without having much insight into the actual experiences of the people occupying the building. Comfort level can impact people’s well-being and therefore it is vital to provide people with a mechanism through which they can have their say. Occupants who experience over-heating or over-cooling problems in such environments with no control over the HVAC settings rarely have a way to provide feedback to initiate corrective action other than calling the facilities management team and informing them of the problem.

To add to this, occupants in different areas of a campus tend to change throughout the day. Students move from one classroom to another depending on their lecture schedule. Their comfort level across these different classrooms may be different. The tolerance of people to cooling/heating levels varies. Whilst it may not be possible to please everyone, at least there could be opportunities to identify an optimum set point that would be comfortable for the majority whilst at the same time reducing the cooling/heating requirement and thereby reduce energy consumption. The work described in this paper is a step in that direction.

In this paper, we make the following contributions: (1) We elaborate on an experimental study comprising a proof of concept IoT-based system designed, developed, and deployed in a number of classrooms across several buildings on a university campus; (2) A cross platform smartphone application designed and developed for both the Android and iOS platforms to facilitate occupant engagement anonymously; (3) A prototype system for fine-grained temperature and humidity measurements and occupant feedback on their perceived thermal comfort and lighting level and their mood in the premises from where they provided feedback; (4) A novel data-driven dashboard for the facilities managers; (5) Insights from the collected data that highlight opportunities for enhancing occupant comfort and well-being and for reducing cooling levels which have the potential to deliver substantial savings taking into account the cumulative savings effect. The proposed solution is low cost and easy to deploy and has the potential to reap significant savings.

The rest of the paper is organised as follows: Related work is reviewed in Section 2. We provide details of the design, development, and deployment of our proposed solution in Section 3 following which we elaborate on the findings from a pilot deployment in our university campus in Section 4. We then discuss some of the challenges in Section 5 and conclude with a summary of findings including pointers to directions for future work in Section 6.

## 2. Related Work

In this section, previous efforts on comfort and well-being and comfort monitoring are elaborated upon.

### 2.1. Prior Work on Comfort and Well-Being

The UAE typically has high temperatures reported in most months of the year with temperatures above 45 degrees Celsius being touched in the summer. In order to ensure that the outside heat does not affect the occupants, the temperature is typically regulated indoors using air conditioners. Most of the buildings tend to have a Building Management System (BMS) which is centrally controlled.

This can have implications on occupant comfort and satisfaction due to a lack of direct control. Environments with no individual control over the temperature can result in health issues. Neck pain, back stiffness, low concentration, nose bleeding, and low levels of Vitamin D are all a result of an indoor lifestyle that minimises exposure to sunlight and fresh air [1].

Comfort and happiness are two terms which are commonly used to define well-being. The best way to define the term comfort is through measurable parameters of the environment and their correlation with the comfort reported by the occupants [2]. When occupants have more control over lighting and temperature, it improves their sense of physical and psychological comfort and well-being and also leads to lower energy consumption [3]. Happiness on the other hand is defined as emotions experienced when in a state of well-being which can range from emotions of contentment to intense joy [4].

According to the global happiness policy report [5], there is a significant amount of evidence showing that people’s work performance is better when they are happy. Work plays an important role in a person’s life where people tend to spend one third of their waking hours and work place quality influences the well-being of workers [6]. This stresses upon the need for programs and initiatives directed towards promoting well-being in the workplace. Keeping this framework while recognizing how integral working in the field of well-being is in the United Arab Emirates, the current study was directed to provide data driven insights on how thermal comfort can have an impact on a person’s overall well-being. Existing studies have focused more on the infrastructure aspect and energy consumption rather than exploring the concept of well-being. With growing awareness on the concept of well-being, many people are demanding better indoor environments that will enhance their well-being and have a positive impact on their productivity and work quality. This further stresses the need for data covering psychological and social dimensions which previous studies ignored as they focused more on the concept of sustainability. The aims of this study have been to explore both sustainability as well as the well-being angles.

People spend almost 80–90 percent of their time indoors and many of their health effects are determined by building characteristics, lighting, ventilation, and indoor environments [7,8]. A study conducted by Karjalainen [9] concluded that people tend to feel hot and cold more often in offices than in homes both in winters and summers. In office environments, occupants generally struggle with having adaptive opportunities which are easier at home such as wearing something warm which is hard in the office due to a strict dress code or even when moving from one room to another [9]. Moreover, low knowledge of heating and ventilation systems coupled with a temperature preference conflicting with the other occupants’ preferences are all major obstacles for people suffering in such an environment, unlike at home where it is easier to manage the optimal temperature.

Several studies have shown organizational environments such as indoor lighting and indoor temperature to have an impact on a person’s mood and well-being [10,11]. Research states that the psychological effects of light on an individual include affecting their feelings, behaviour, and performance. Feelings refer to the subjective experience of how a person feels under the lighting condition, behaviour refers to the influence the lighting condition has on a person’s actions, and performance concerns how good or bad a person can perform at their work [12]. With the structure of many buildings, many workplaces are deprived of daylight. With a minimum of eight hours of work per day, the need for optimal lighting and temperature in these office settings is extremely important [11]. Studies have shown that bright polychromatic light during daytime increases alertness and cognitive performance [11]. Moreover, during winter it is seen to enhance mood and improve vitality amongst workers [13,14,15]. There is a significant amount of evidence which suggests that the brightness of ambient light is not only important for the completion of tasks but is also seen to impact alertness, improve health related quality of life, improve productivity, and alleviates distress [16]. Thus, the importance of ambient lighting on mood and its overall effect on occupants’ well-being needs to be taken into account.

### 2.2. Prior Work on Comfort Monitoring

A wireless sensor test bed to monitor the comfort level in a two-century old building is proposed in [17]. They used Java enabled VirtualScene node enablers integrated with sensors, micro-controllers, and transceivers in the test bed. Numerous external sensors present in the node enabler perform multi-tasking such as people counting, measurement of the noise, carbon dioxide level, temperature, light, and pressure. A similar approach is proposed in [18] which comprises nodes measuring various metrics such as temperature, humidity, lighting conditioning, sound level, and people occupancy in a building. A plug and play learning framework is proposed in [19] to identify the thermal model of each thermal zone in a building without any manual configuration to control the energy consumption of many electrical devices fitted in large buildings. The proposed thermal model learns from the data provided by IoT-based smart thermostat sensors. Kannan et al. in [20] have proposed an energy management system (EMS) to reduce the cooling load demand (CLD) in large buildings by fixing an optimal thermal zone temperature set point using fuzzy concepts. They used a hybrid approach with an Artificial Neural Network (ANN) model to predict the zone temperature for next time slot and regression approach to model zone cooling energy behaviour to predict zone CLD for the next time slot. To obtain the feedback, the authors further used a zone thermal feedback model and estimated the comfort margin of the occupants based on thermal conditions and predicted mean vote index. Aryal et al. in [21] have implemented intelligent agents to measure the thermal comfort zone of every individual occupant and control their personal comfort system, e.g., fan, heater. Whilst this was based on sensing and actuation of individual devices, this can be challenging in shared spaces where HVAC systems cater to large numbers of people and cannot be tuned for individual comfort. Hu et al. [22] proposed the use of environmental data and data from wearable devices for vital sign monitoring (a proxy for thermal comfort). They collect, process, and manage the data using cloud and machine learning models for modeling the thermal comfort. Whilst introducing wearables in the mix is certainly an interesting angle, this approach can add to the cost and may have potential privacy implications.

Dimara et al. [23] have used statistical approaches to measure the optimal indoor comfort conditions of the occupants in residential houses of two European countries. Temperature, humidity, and luminosity sensors were installed in each residence to collect the environmental data and feedback was collected from the occupants regarding their experiences with indoor temperature and visual comfort to identify the optimal set point.

Amaxilatis et al. [24] explored the use of an IoT-based system in school environments for encouraging positive user behaviour changes towards energy efficiency. This was accomplished by deploying their system and running healthy competitions to nudge user behaviour. Park et al. [25] utilised a text mining approach to analyse literature on thermal comfort and building control research. They highlighted an increased focus on controlling a building’s energy consumption in comparison to occupant’s thermal comfort. Jayathissa et al. [26] introduced a controlled study in gathering thermal, noise, and acoustic preferences involving thirty occupants in an office building. Their findings highlight that by integrating the feedback with environmental, physiological, and historical preference data, they were able to predict occupant preferences with an accuracy of up to 86 percent. Peng et al. [27] present a learning-based demand-driven control approach for cooling in office spaces. They show around twenty percent savings compared to their baseline by predicting the occupants’ presence and their time spent in the premises and utilizing this information as occupant behaviour to adjust the temperature set points.

Based on the above, we observe a variety of approaches (ambient sensing, data from wearables, occupant feedback, machine learning, etc.) to understand thermal comfort of occupants in buildings some of which are geared towards identifying the optimal set point. As highlighted in Section 1, achieving this in a shared setup where occupants change continuously and where they may not have direct control is much more challenging. It is this aspect that our work primarily focuses on—the objective being not only to assess occupant comfort and identify opportunities for energy savings but also to explore the effect of other parameters on the happiness and well-being of the occupants.

In our earlier work [28], we proposed a preliminary prototype using the Arduino platform, a basic version of an Android app, and early results from a small scale study. Subsequently, work has focused on (1) migrating to the Raspberry Pi Zero platform (since it consumes much less power), (2) automation for ease of large scale deployment and real time asset status tracking, (3) extending the scope of the initial version of the app to include additional items such as capturing information on the lighting levels and mood related parameter in addition to thermal comfort for assessment of people’s well being, (4) development of the app from scratch using Flutter to enable an application for both the Android and the iOS platforms, and finally (5) deployment and pilot trial of the solution involving people on the UAEU campus at scale. We now elaborate on the proposed solution and findings from our study.

## 3. Proposed Solution

This section provides details on a variety of aspects of the proposed solution such as the system architecture, design, data processing, deployment, and pilot trial. The proposed solution consists of two parts—a hardware prototype for gathering fine-grained data from the premises being monitored and a smartphone app to enable occupants to provide feedback on their experience in the premises. Unless stated otherwise, in the remainder of this paper we will refer to these as the ‘Cozy System’ and the ‘Cozy App’, respectively. We also use the term ‘Cozy Solution’ to refer to the complete solution encompassing both of the above.

### 3.1. Cozy System

**Figure 1:**
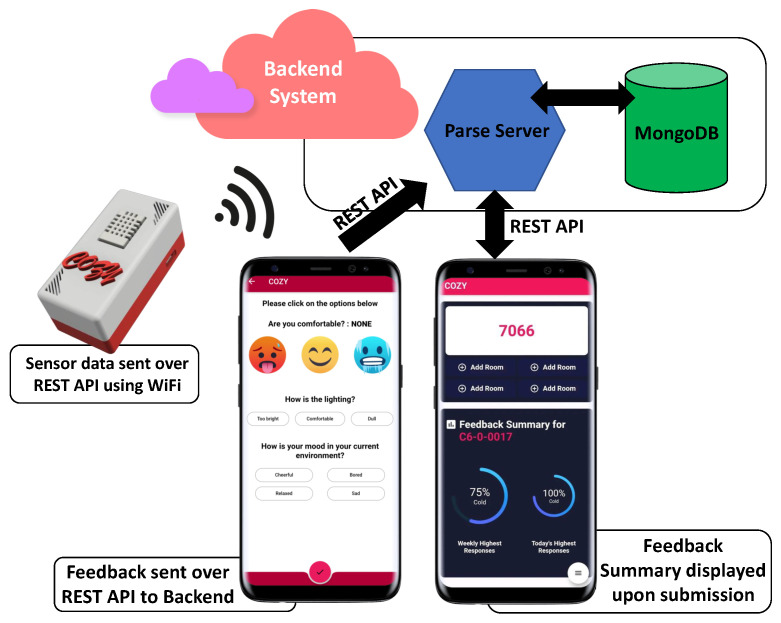
High level system architecture.

The Cozy system was realised using off-the-shelf components, Raspberry Pi Zero (hereinafter referred to as ‘Pi’), and a DHT-22 sensor which measures temperature and humidity. The on-board WiFi module on the Pi in each prototype box was used to connect to the university WiFi network in order to transport the sensor data from the premises where the box is deployed to a backend system. Numerous Cozy boxes were deployed in different locations on the campus to gather fine-grained data from these locations. Figure 1 shows the high level architecture of the Cozy system.

**Figure 2:**
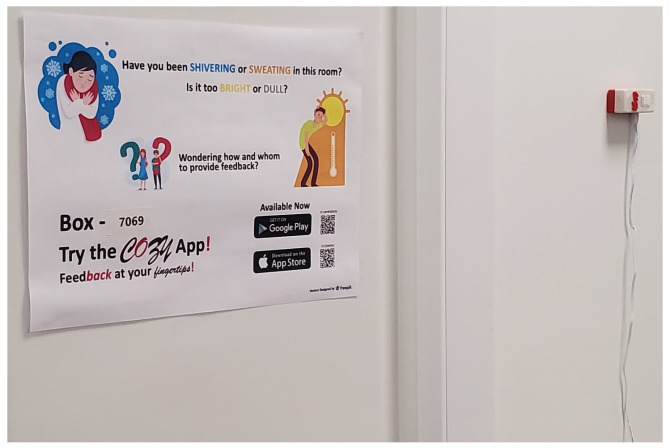
Cozy box deployed in a classroom.

Additionally, to ease deployment and maintenance at scale, an asset management system was designed and developed. The UAEU campus has a QR-based location tagging system for each of its rooms. There is a unique QR-code associated with each room which manifests as a QR-code sticker on the entry door for that room. We provided each Cozy box with a unique box ID during the initial setup process. We utilised the above-mentioned QR codes for associating the deployed box to its location. We developed a smartphone asset management application which takes as input the box ID and upon scanning the above mentioned QR-code (using the smartphone camera) at the location where the box is deployed, maps the deployed box against this location in the backend system. This way, the deployed assets (Cozy boxes) can be tracked accurately even for a large-scale deployment. Each box was configured to capture fine-grained temperature and humidity measurements (interval configurable from the backend system) and reports this along with the WiFi signal strength and the timestamp to the database. Figure 2 shows one of the Cozy boxes deployed in a classroom.

### 3.2. Cozy App

**Figure 3:**
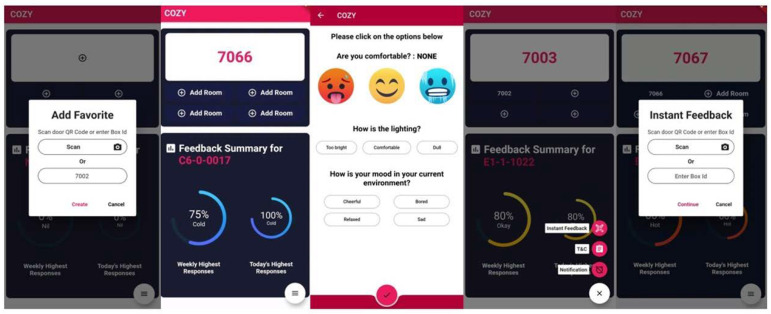
Screenshots of the Cozy application.

As highlighted earlier in this paper, it is important to know the ground truth, i.e., the comfort level experienced by people at their location. To facilitate gathering such information, a smartphone application was developed using Flutter. Flutter is an open-source application development framework that can be used to build natively compiled apps across multiple platforms using a single codebase. Since the app was to be used across a widespread audience, we decided to make it available for both Android and iOS platforms. Figure 3 provides an overview and some sample screenshots from the Cozy app.

Posters on how to use the Cozy app were posted in each location where a Cozy box was deployed (see Figure 2). In the first step, the occupant can download the app from their respective application store using the provided QR-code (shown on the poster in Figure 2). Once downloaded and installed, the occupant does not need to log in as the application reports data anonymously. The occupant then has the ability to add their most frequented location in the application homepage for providing feedback or utilizing the instant feedback option. Assuming a student typically takes five courses in a semester, space for five favourite locations was provisioned on this page. The occupant needs to click on ‘Add Room’ for adding a location to their favourites. Upon selecting this (see the first screenshot in Figure 3), they can either scan the QR-code found on the room door or enter the box ID found on the Cozy box. For ease of access to provide feedback, the box ID was also shown on the poster displayed in the room (see Figure 2). Clicking ‘create’ subsequently adds this room as one of their favourites. This is a one time process that the occupant needs to do in each of their favourite locations.

Selecting a box number by clicking on it brings it to the top of their favourites list and displays the corresponding room number in the bottom half of the screen along with a summary of all responses for that location (second screenshot in Figure 3). To provide feedback, the occupants need to click on the box number which launches the feedback page (third screenshot of Figure 3). As shown on this page, there are three simple questions the occupant needs to answer—one about their thermal comfort (whether they are feeling Hot/Comfortable/Cold) by clicking on the corresponding emoji, their perceived lighting level, and their mood in the current environment. Clicking the ‘tick’ at the bottom of the page sends the feedback data to the backend system and takes them back to the home page of the app where they can see the summary responses for this room based on the feedback data (contributed by them and others) for this room. There is also the option to provide instant feedback (without adding a room). For example, if a student is visiting a non-frequented location and wants to provide feedback (perhaps, one time only) on their comfort level at that location, they can click on ‘Instant Feedback’ (see fourth screenshot in Figure 3. This launches the page shown in screenshot five of Figure 3 wherein the occupant merely needs to scan the location QR-code or enter box ID and answer the same questions as above.

Building on prior experience of conducting smart parking pilot trials using a smartphone application [29], the Cozy app was designed bearing in mind the need to simplify the user interface and minimise user interaction. Users confirmed the intuitiveness and user-friendliness of the Cozy app interface during offline conversations.

### 3.3. Cozy System Backend and Data Processing

The Cozy system backend is composed of a MongoDB instance running on a local Linux server. The Parse platform [30], which is a Backend-as-a-Service platform, was integrated alongside MongoDB to manage the server side infrastructure. Interfacing between the monitoring device (Cozy box) and the MongoDB instance in the backend was facilitated with the help of the Parse platform’s built-in features. The Parse platform uses Java Script Object Notation (JSON) as its main data format which makes it easy, fast, and efficient to store and retrieve data from MongoDB. Parse also provides a built-in Software Development Kit (SDK, version 1.4.0) and simple to use Representational State Transfer (REST) Application Programming Interfaces (APIs) which can be used for communicating with the database.

Temperature and humidity data from each Cozy box were continuously logged into the database on a ten minute interval. This interval is configurable in the backend and can be changed on the fly. When the occupant provides feedback corresponding to their location using the Cozy app, the app queries the most recent temperature and humidity data from the database for that location. This consolidated information (timestamp, location, temperature, humidity, occupant feedback) is then stored as a feedback entry in the database.

The backend scripts were designed to support specific functionalities in the Cozy system. One of the scripts runs in the background checking the status of each of the deployed boxes and flags an alert in the event that a box needs attention; for example, a power outage or poor WiFi signal which prevents the box from reporting data. Another script computes summary statistics on a daily and weekly basis by aggregating the feedback data for each location and updates this in the database corresponding to this location. When an occupant gives feedback using the Cozy app, the above-mentioned summary data is fetched from the database and displayed on the occupants’ screen. This is to help the occupant gain insight in terms of their perception and the perception of others about this shared space. Feedback provided by the occupants was used for understanding and determining potential temperature set points that can meet the comfort of the majority of the occupants keeping in mind the need to reduce energy consumption.

### 3.4. Methodology, Deployment, and Pilot Trial

To evaluate the usefulness of the proposed solution, forty-one rooms in five buildings on the UAEU campus were instrumented with the Cozy box (see an example in Figure 2). Of these, six rooms in two of the hostel buildings were single occupancy rooms and five rooms were in the shared labs building E3. The remaining rooms were in the E1 and the C6 buildings. In some of the rooms, the box was deployed on the wall (as shown in Figure 2) whereas in some other rooms, it was deployed on the podium in the room. Feedback was collected anonymously between January 2021 up to February 2022. However, because of COVID-19-related restrictions highlighted in Section 5, gathering regular feedback was a significant challenge. Since the feedback collected is anonymous, it is not possible to identify the individual user who gave the feedback. It is possible to identify the location from where data is gathered since the infrastructure data (box measurements) as well as the feedback data (from the Cozy app) is linked to a specific location. Classrooms are separated for male and female sections within the UAEU campus and therefore, depending on the location from where data is gathered, it is possible to identify the link between each feedback and gender. The majority of the respondents were University students in the age bracket of 18–25 years.

Each of the Cozy boxes were configured to report data on ambient parameters on a ten minute timescale to the backend system. Information about the Cozy app was advertised through internal communication to students and staff. The Cozy app was made available for anyone within the university to download from the Google Play store and the Apple App store. Flyers were also posted in the rooms where the Cozy box was deployed containing instructions on how to download and use the app. The Cozy project team visited numerous classes to give a demo on how to use the app and to encourage everyone to provide feedback as much as possible. Lecture instructors were also encouraged to nudge students during the class to provide feedback using the Cozy app. The feedback collected by the Cozy app was anonymous and targeted towards assessing the comfort levels of the occupants.

The inclusion criteria for the participants in this research were the occupants of the room where the Cozy prototypes were installed. Ethical approval was obtained from the University Ethics Committee and participants were ensured that all data received would be kept anonymous and no harm will be caused by the prototypes that were installed in the rooms. Proper explanation of the structure of these prototypes was provided to the participants and their questions were addressed. Also, the participants were assured that their participation is voluntary and they can choose to withdraw from the research at any point in time.

Within the Cozy application, three questions on temperature, lighting, and mood were included to assess occupant comfort, potential for enhancing building sustainability, and well-being. Considering the barrier of not having many questions that could restrain the participants from giving regular feedback, the questions were kept to a minimum. The first question assessed the comfort level in which responses were collected by three emojis portraying hot, normal, and cold temperature. The majority of the respondents were university students between the age bracket of 18–25 years and engaging this audience to seek regular feedback was challenging. Therefore, emojis were used to not only make giving feedback convenient but also to grasp the participant’s attention [31]. Studies have highlighted that younger generations show greater preference for using emojis to deliver their answers as it is quicker and is seen to improve data quality and survey evaluation [31]. The second and third question assessed the lighting and mood on a three and four point rating scale, respectively. The second question ‘how is the lighting’ had three options ‘Too bright, comfortable, and dull’. These three options were chosen to assess the light preference based on prior studies that were conducted to evaluate the optimal lighting required to assess the work performance. The third question assessed the mood as ‘cheerful, bored, relaxed, and sad’. All these four categories were chosen to be based on concrete data to not confuse between mood and emotions as we were assessing mood only.

## 4. Results

This section highlights the observations from the pilot trial of the proposed solution and summarises the key findings from this study.

### 4.1. Occupant Comfort and Energy Conservation

**Figure 4:**
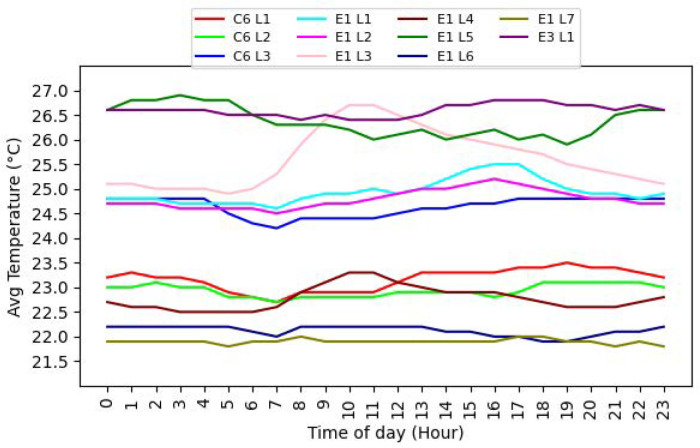
Temperature at different indoor locations.

Figure 4 shows the average temperature at the different indoor locations in the UAE university campus. In the vast majority of the cases, the variability at each location is within a 0.5 °C range although the temperatures at some locations varies significantly. As an example, we notice one of the rooms (E1 L7 in Figure 4) consistently operating at 22 °C, another one (E1 L2) operating at 24.5±0.5 °C, and E1 L5 operating between 26–27 °C in the same building.

**Figure 5:**
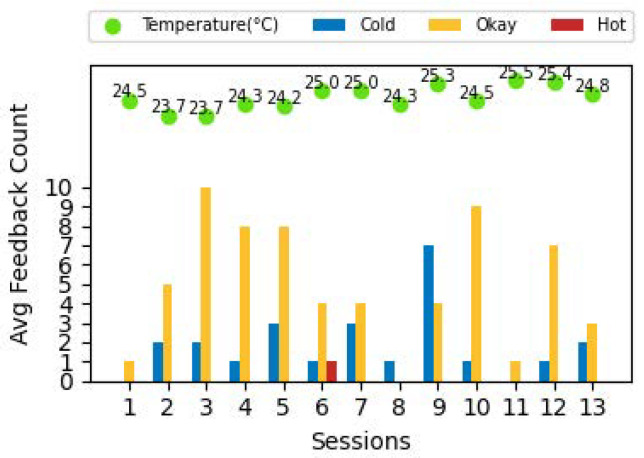
Feedback from a classroom in building C6.

Figure 5 shows the feedback obtained in one of the classrooms in the C6 building over a number of sessions during a semester. This feedback was provided by the students attending the same course at the same time on two different days of the week. The green dots overlaid on the bars indicate the average temperature recorded in the classroom during the session. Observe that in the vast majority of the cases the occupants are comfortable in this room. The temperature range for the ‘Okay’ feedback varies from 23.7 to 25.5 °C. Given that the majority of occupants are comfortable at a wide range of temperatures, from an energy efficiency perspective, it may be prudent to set the temperature to a higher set point in this case which would mean a reduced need for cooling translating to a corresponding decrease in the energy consumption, associated cost, and CO_2_ emissions.

**Figure 6:**
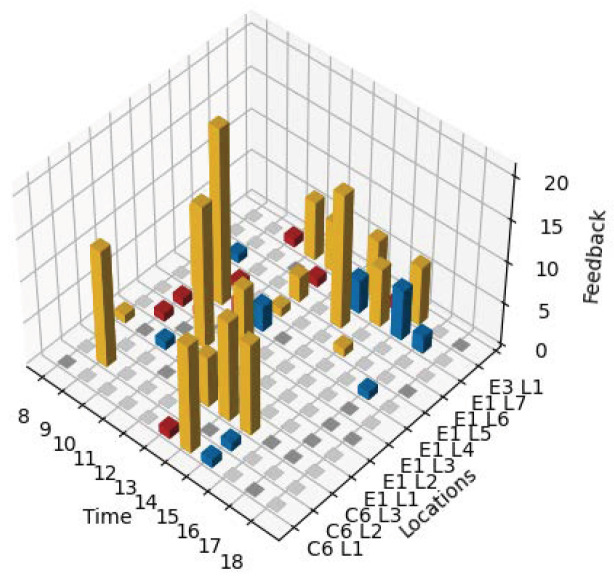
Variation in occupant comfort at different times across different locations.

Figure 6 shows the occupant feedback at different times across different locations. For each location, the bar represents majority feedback during the hour. The amber, red, and blue bars indicate the occupant feedback to be comfortable, hot, and cold, respectively. The empty spaces indicate hours when no feedback was received. This could be either due to no classes taking place at the location during that time or no feedback being received. As evident from Figure 6, we observe varying comfort levels in the different locations and also varying comfort levels at the same location at different times. In general, whilst the majority of feedback data in Figure 6 indicates that the occupants are comfortable, there are several locations where the occupants reported being cold. For example, in the case of location E1 L7, there are several feedback points indicating cold. This could potentially be attributed to a low average temperature at this location (around 22 °C as seen from Figure 4). The varying feedback at different times at the same location could be attributed to occupants varying across the same shared space. We also observe that there are very few locations where the occupants reported being hot. This seems to suggest that over-cooling is relatively more dominant than the overheating problem. Overall, the observations from Figure 6 reinforce that a single setting may not be the best choice at all times and that there is a need for regular reassessment and adaptation.

**Figure 7:**
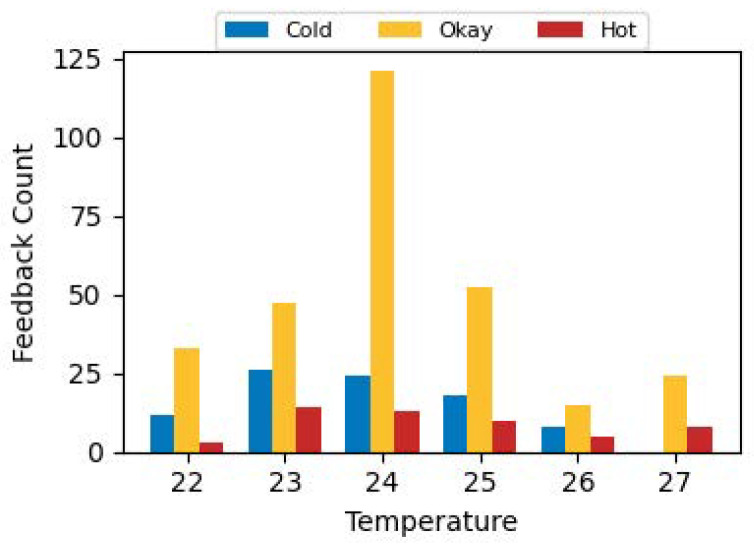
Feedback vs. Temperature.

Figure 7 shows the feedback obtained corresponding to a range of temperatures between 22 to 27 degrees for the different deployment locations. Whilst the majority response for each temperature is comfortable, the lowest fraction of responses indicating some issue (either hot or cold) corresponds to a setpoint of 24 °C. Such data driven insights could help facilities managers identify energy efficiency settings with minimal impact on occupant comfort.

**Figure 8:**
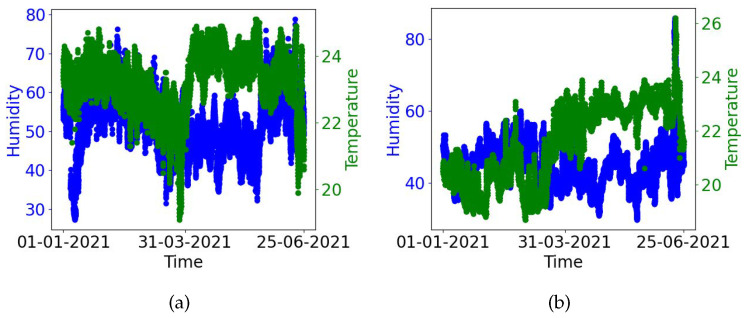
Benefit of the Cozy system (pre-intervention and post-intervention cooling). (**a**) Location 1, (**b**) Location 2.

Figure 8 shows an example of the benefit of the proposed system (relying on infrastructure measurements) for a couple of rooms in the CIT building. The figures show the temperature (green points) and humidity (blue points) in these rooms. As evident from these figures, the average temperature range in these rooms in the first half of both figures was on the lower side. The facilities management team was subsequently notified about this which triggered a control resulting in a subsequent reduction in cooling in these premises as evident from the temperatures in the second half of these figures. As it stands, only aggregate level building energy consumption data is captured by the facilities management team with no infrastructure support available for sub-metering individual locations and connected loads. This building comprises a variety of locations such as classrooms, faculty offices, meeting rooms, auditoriums, computing labs, IT equipment rooms, student common rooms, and large open spaces. Given the lack of sub-metering, it is difficult to quantify the precise energy savings that could be achieved with the Cozy pilot deployment. Publicly available estimates such as [32,33] indicate that around 5–10 percent energy could be saved through increasing the temperature set point by a degree. In comparison to these, even if we are to assume relatively conservative savings percentages from deployment of the proposed solution, this could potentially translate to substantial savings over a large number of rooms/buildings over a long period of time. In the context of this study where the majority of our boxes were deployed, the premises are cooled by fans circulating cold air from the chill water fed from a chill water plant. Energy is used during the production of chilled water, by the pumps circulating the chilled water through the pipes, and by the above-mentioned fan units which circulate the cooled air into the rooms. Cooling in this building can be tailored by adjusting the fan speed through the building management system (BMS). The fan speeds can be set to low, medium, or high mode to regulate air flow. Assuming an energy consumption of 3 kWh, 4 kWh, and 5 kWh per day in these three modes and that fan speeds at each mode result in a degree Celcius difference in the room temperature, a reduction in cooling by switching to a lower mode could potentially yield 1–2 kWh/day per fan unit at a location. There are numerous such fan units in this building (100+). Even if we are able to reduce cooling by a degree or two at half of these locations, this could yield savings in the range of 50–100 kWh/day on average. Accumulating such savings over a large number of locations could yield substantial savings over time.

### 4.2. Operational Dashboard for Facilities Managers

**Figure 9:**
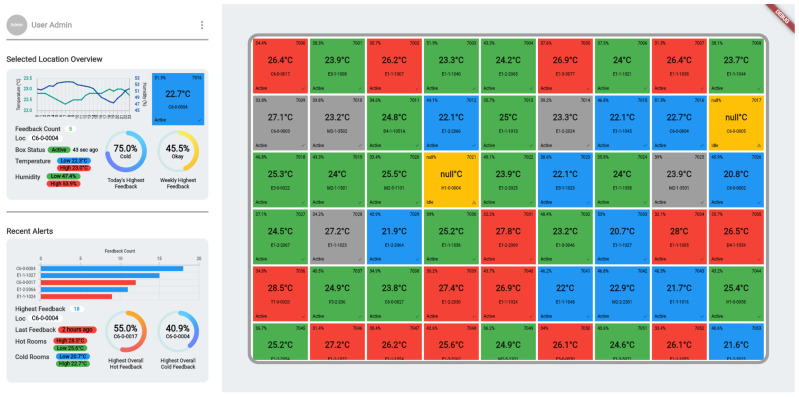
An example of a Dashboard for facilities managers.

In addition to providing insights for the occupants on how others are feeling about the room (bottom half of the second screenshot in Figure 3), it could also be beneficial to provide insights for the facilities managers to help them identify rooms that require their attention even before occupants start complaining. Figure 9 shows an example of a dashboard under development (not yet deployed) for the building manager which relies on both infrastructural measurements such as those provided by the Cozy box and on occupant feedback. Each cell in the dashboard shows the location information, status of the box in that location, and values of temperature and humidity at the location. These are based on the real data collected from these locations. The colour code of a room is supposed to visually inform the building manager whether occupants in a room are Hot (Red), Cold (Blue), or Comfortable (Green) based on their feedback (currently based on synthetic data which in future could use actual feedback data). It should be noted that this would be based on summary statistics over multiple feedback data points rather than an individual one. For example, if the majority of responses over a time window indicate ‘Cold’, the cell corresponding to this location would be coloured Blue. A grey cell indicates absence of feedback from a location which could prompt the facilities manager to adjust for a lower cooling in order to maximise energy savings and an amber coloured cell indicates a box that is not reporting data. Selecting a cell shows the summary statistics for that location as seen on the block on the top left of this figure. Such a system would offer the potential for the facilities manager to engage in proactive management for timely resolution of over-heating/over-cooling issues facing the occupants. Whilst traditional systems may have access to infrastructural measurements, in the absence of occupant feedback, they will not be able to know if there is a problem. The proposed solution could therefore be a tool to empower both the occupants as well as the facilities managers.

### 4.3. Occupant Comfort and Well-Being

**Figure 10:**
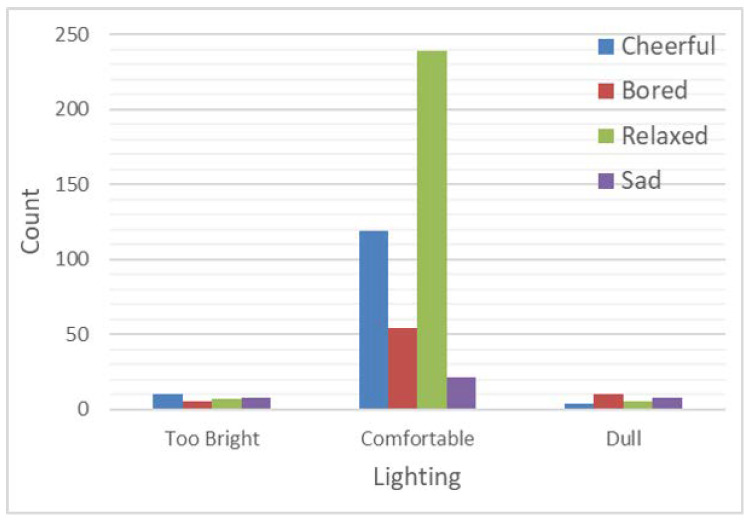
Relationship between lighting and mood.

Figure 10 and Table 1 show a strong association between mood and lighting with a value *p* < 0.001. Crosstabs applied as mood and lighting were both categorical variables indicating that mood is significantly associated with lighting. With the results of this table, we can observe that the highest percentage of relaxed mood was reported (55.2%) in comfortable lighting. These results can further be supported by research and studies which have shown a strong relationship on the effect of lighting on mood such as the studies by Kuijsters et al. [34] and Kong et al. [35]. According to the results of our current study, high percentages were reported on boredom and sadness for dull lighting which can be confirmed by the previous research on how dull lighting would reduce the participant concentration and attention thus leading to boredom [36].

Table 2 shows a strong association between mood and lighting across both male and female gender with a value *p* < 0.001 (significant). Males have reported being more cheerful (37.5%) at too bright light compared to females (30.8%). This finding can be supported by the study conducted by Belchar et al. [37] which highlighted that mood shift was negative for females in bright light compared to males, whereas males responded in the opposite direction. In comfortable lighting, more females reported having a relaxed mood (57.9%) than males (45.9%). This can be supported by a study stating that females were significantly higher in positive mood than males [38]. Sad mood was seen to be reported more in females (33.3%) in dull lighting compared to males (22.2%). This finding can be supported by research conducted on Seasonal Affective Disorder, a condition characterised by seasonal depressive episodes primarily affecting individuals away from the equator and having less light. This disorder is seen to be reported more in females than in males and low mood is one of the main conditions of this disorder [39]. Although the UAE topography has adequate bright light, temperatures hitting high forties (°C) are a major obstacle for people going out and engaging in outdoor activities. The aforementioned finding could be explained by the infrastructure of the buildings where the study was conducted with rooms within certain locations of the campus having limited access to sunlight and indoor lighting was also minimal. This could be a cause of the sad mood being reported, especially in females due to their minimal exposure to sunlight and limited movement outdoors. We also performed the analysis to assess the association between temperature and gender but the results were not statistically significant.

In summary, the following are some of the salient findings from this study: A fixed thermal operating set point may not be optimum for all occupants in the building. In light of the variability of occupants in different shared spaces at different times of the day, regular reassessments and adaptation of the set point is vital. Furthermore, in the majority of the cases wherein occupants are comfortable at multiple different set points, choosing a higher set point amongst these yields opportunities for reduced cooling and energy savings. Whilst traditional building management is reactive in nature and primarily driven by infrastructure measurements as proxy for occupant perception, the proposed approach provides opportunities for occupant engagement and can help to flag over-cooling/over-heating issues early on which can facilitate proactive management. Therefore, this approach empowers both stakeholders. Finally, with respect to the correlation between lighting and mood, our findings are in line with previous studies.

## 5. Discussion of Challenges and Limitations

In this section, we highlight some of the challenges and limitations associated with this work and potential ways to address these.

Given the lack of a student population on-campus due to COVID-19 restrictions, it was difficult to obtain student feedback. Although we did not obtain occupant feedback data, we were still obtaining the temperature and humidity data reported by the Cozy box from the deployment locations. Based on this data, we were able to identify several rooms that were over-cooling. The facilities management team was made aware of this which resulted in positive action resulting in a reduction in cooling.

Subsequently, when the alternating online/offline model of campus access came into play, some data collection was possible. Not being on-campus on all days posed a significant challenge to occupant engagement as people tend to forget using the app. We observed that unless reminded, it was hard to collect feedback data from the occupants. In some cases where the occupants experienced discomfort (hot/cold), feedback data was received. This is unsurprising because people typically tend to give feedback only when they are unsatisfied with something. Most of the time, they will not bother giving feedback if there is not a problem. However, as highlighted from one of the findings in the results section, obtaining feedback even when things are fine can be really useful, e.g., to identify the highest temperature where the majority are comfortable which can yield a degree or two of reduction in cooling. As highlighted in the last paragraph in Section 4.1, this has the potential to achieve substantial energy savings over many locations over time.

Two potential approaches could be experimented with in the future to address the aforementioned issue—(1) appropriate messaging to show the occupants’ contribution because of the feedback they provide, e.g., ‘your feedback helped reduce cooling saving X amounts of CO_2_ emissions and shaved the energy bill by Y’ and (2) setting the operating points a degree or two above the ‘status-quo default’ and thereby aiming for reduced cooling by design. If this would make people uncomfortable, they would provide feedback which can then feed into corrective action. If no feedback is received, this would imply people find the current settings acceptable which would lead to energy conservation by default.

Another major challenge we encountered was the proprietary HVAC system used in the campus which made it difficult for third party control. Any actuation or changes have to go through the system either triggered by the BMS operator or through integration with the existing system. Such integration is expensive and challenging because the system integration has to go through the vendor. This necessitates an open solution. Secondly, the type of cooling system in place also has a significant impact on what is achievable. For instance, when using a chill water plant for cold air circulation, fine grained adaptation may not be feasible as the chill water plant cannot be turned on and off on short time scales. The ability to have variable frequency drives that can tailor air flows on demand can be beneficial in such cases but the cost benefit analysis has to be established before the relevant stakeholders can be convinced for an uptake.

One of the findings from this work was a lack of statistically significant association between temperature and gender. This is different from the findings reported by Karjalainen [40] which showed higher sensitivity of women to their thermal environment in comparison to men. This inconsistency could potentially be attributed to the local cultural clothing norms and lifestyle. Given the hot climatic conditions, there is a significant difference between indoor and outdoor temperatures with people living mostly in air-cooled environments. The vast majority of the occupants on campus are students from the UAE, both male and female students, following the traditional national dress code which covers the full body. This might have affected their responses. Secondly, any kind of monitoring is likely to be met with skepticism, in particular, worry about what is being monitored and what the data would be used for. On many occasions, especially the female participants were apprehensive about whether our prototype had a camera or a voice recorder within, which was clearly not the case. This could have impacted their responses which might be a possible explanation for the insignificant association between temperature and gender observed in the current study.

## 6. Conclusions

Buildings in geographies with hot climatic conditions have a significant environmental footprint with systems for cooling being typically threshold driven and not taking into account the needs of the changing occupants especially in shared spaces such as classrooms on university campuses which lack direct control. In this paper, we elaborated on the design and implementation of a proof of concept solution called ‘Cozy’ for addressing this issue. The Cozy system combines ambient measurements (sensing infrastructure) with occupant feedback (solicited from the occupants using a smartphone app) to provide insights to the building manager for enhancing thermal comfort. Findings from a pilot deployment on a university campus using the proposed solution have shown promise for the proposed solution.

In particular, we showed that the occupant experience varies across different times at different locations which motivates the need for regular reassessments especially in shared spaces with a centrally controlled HVAC. It is possible to optimise for energy efficiency and at the same time balance thermal comfort by choosing the highest set point (least cooling) at which occupants are comfortable with estimates indicating a 50–100 kWh/day of potential energy savings per building. We also showed how to identify and reduce excessive cooling through infrastructural monitoring as a result of deployment of the Cozy system. A notable highlight is the dashboard for facilities managers to enable proactive management as it can help them identify locations needing their attention even before they start receiving complaints. Finally, we also evaluated the impact of lighting on the mood across genders based on the occupant feedback data and found a strong correlation between these. Since occupant comfort and well-being has an impact on their productivity, it is vital that the ambient environment be conducive to this. Similar to how cooling is vital in countries exhibiting a hot climate, heating is vital in colder geographies and has a corresponding contribution to the overall energy footprint. Whilst the study in this paper focused on the former, it would be interesting to carry out a similar study on the latter.

Future work could also look into strategies for enhancing occupant engagement, fine grained metering to assess precise energy savings resulting from the interventions, exploring ways to facilitate adoption of the proposed dashboard to augment the capabilities of the existing BMS system and carrying out cost versus benefit analysis with and without using the proposed system.

## Figures and Tables

**Table 1:** Cross tabulation of mood across lighting.

Lighting	Cheerful (n)	Cheerful (%)	Bored (n)	Bored (%)	Relaxed (n)	Relaxed (%)	Sad (n)	Sad (%)
Too bright	10	33.3	5	16.7	7	23.3	8	26.7
Comfortable	119	27.5	54	12.5	239	55.2	21	4.8
Dull	4	14.8	10	37.0	5	18.5	8	29.6

**Table 2:** Cross tabulation of mood and lighting across gender (M: Male, F: Female).

Mood	Too Bright M (n, %)	Too Bright F (n, %)	Comfortable M (n, %)	Comfortable F (n, %)	Dull M (n, %)	Dull F (n, %)
Cheerful	(6, 37.5%)	(4, 30.8%)	(32, 29.4%)	(87, 27.1%)	(1, 11.1%)	(3, 16.7%)
Bored	(5, 31.3%)	(0, 0.0%)	(17, 15.6%)	(37, 11.5%)	(6, 66.7%)	(4, 22.2%)
Relaxed	(0, 0.0%)	(6, 46.2%)	(50, 45.9%)	(186, 57.9%)	(0, 0.0%)	(5, 27.8%)
Sad	(5, 31.3%)	(3, 23.1%)	(10, 9.2%)	(11, 3.4%)	(2, 22.2%)	(6, 33.3%)

## Data Availability

The data collected during the course of this study belongs to the UAE University. This data could be made available on request subject to approval from the University.
